# Prospects and limitations of cumate-inducible lentivirus as a tool for investigating VEGF-A-mediated pathology in diabetic retinopathy

**DOI:** 10.1038/s41598-024-63590-y

**Published:** 2024-06-21

**Authors:** Inesa Lelyte, Vidhya R. Rao, Giedrius Kalesnykas, Symantas Ragauskas, Simon Kaja, Zubair Ahmed

**Affiliations:** 1 Institute of Inflammation and Ageing, University of Birmingham, Edgbaston, Birmingham, B15 2TT UK; 2 R&D Division, Experimentica Ltd., 10243 Vilnius, Lithuania; 3 Department of Ophthalmology, Loyola University Chicago, Maywood, IL 60153 USA; 4 R&D Division, Experimentica Ltd., Kuopio, Finland; 5 Experimentica Inc., Fort Worth, TX USA; 6 Department of Molecular Pharmacology and Neuroscience, Loyola University Chicago, Maywood, IL 60153 USA; 7 Centre for Trauma Sciences Research, University of Birmingham, Edgbaston, Birmingham, B15 2TT UK

**Keywords:** Diabetic retinopathy, Cumate-inducible lentivirus, Gene expression, Vascular endothelial growth factor, Retinal toxicity, Cell biology, Drug discovery, Genetics, Neuroscience

## Abstract

Diabetic retinopathy (DR) is a multifactorial disease displaying vascular-associated pathologies, including vascular leakage and neovascularization, ultimately leading to visual impairment. However, animal models accurately reflecting these pathologies are lacking. Vascular endothelial growth factor A (VEGF-A) is an important factor in the development of micro- and macro-vascular pathology in DR. In this study, we evaluated the feasibility of using a cumate-inducible lentivirus (LV) mediated expression of *vegf-a* to understand DR pathology in vitro and in vivo. Retinal pigment epithelial cells (ARPE-19) were transduced with cumate-inducible LV expressing *vegf-a*, with subsequent analysis of *vegf-a* expression and its impact on cell proliferation, viability, motility, and permeability. Cumate tolerability in adult Wistar rat eyes was assessed as an initial step towards a potential DR animal model development, by administering cumate via intravitreal injections (IVT) and evaluating consequent effects by spectral domain optical coherence tomography (SD-OCT), flash electroretinography (fERG), ophthalmic examination (OE), and immunohistochemistry. Transduction of ARPE-19 cells with cumate-inducible LV resulted in ~ 2.5-fold increase in *vegf-a* mRNA and ~ threefold increase in VEGF-A protein secretion. Transduced cells displayed enhanced cell proliferation, viability, permeability, and migration in tube-like structures. However, IVT cumate injections led to apparent retinal toxicity, manifesting as retinal layer abnormalities, haemorrhage, vitreous opacities, and significant reductions in a- and b-wave amplitudes, along with increased microglial activation and reactive gliosis. In summary, while cumate-inducible LV-mediated *vegf-a* expression is valuable for in vitro mechanistic studies in cellular drug discovery, its use is not a feasible approach to model DR in in vivo studies due to cumate-induced retinal toxicity.

## Introduction

Diabetic retinopathy (DR) is the leading cause of preventable blindness in the world and the primary cause of vision loss among working-age adults^1^. DR primarily results in microvascular changes, including pericyte loss^2^, increased vascular permeability^3^ and formation of acellular capillaries^4^. Prolonged duration of diabetes results in the appearance of microaneurysms and haemorrhages, associated with vascular leakage and neovascularization^5^. In the absence of intervention, diabetic retinopathy (DR) may progress to diabetic macular edema (DME) and proliferative diabetic retinopathy (PDR), ultimately culminating in complete vision loss. Animal models which recapitulate vascular pathophysiology of DR are essential for studying and understanding the underlying molecular mechanisms, as well as developing and testing novel therapies to treat the disease.

Vascular endothelial growth factor-A (VEGF-A) is highly upregulated in diabetic retinopathy and induces the progression of various events characterizing the disease, including vascular leakage, neovascularization, and DME^6^. VEGF antagonists are currently in clinical use for treating vascular abnormalities^6^, but such therapies require re-peated intraocular injections. To study the role of VEGF-A and develop novel anti-VEGF-A treatments, VEGF-A-induced animal models have been generated. Intravitreal injections of recombinant VEGF-A have been shown to mimic many of the complex DR mechanisms, including vascular leakage and neovascularization in rabbits^7,8^. However, VEGF-A-induced effects are transient due to the short half-life of injected peptides. To overcome this drawback, gene transfer models which mediate long-term expression of VEGF-A have been established. Adeno-associated virus (AAV) based VEGF-A delivery in mouse model resembled several pathological characteristics of proliferative DR, including vascular leakage, neovascularization, and vitreous haemorrhage^9^. However, existing models do not offer the ability to temporally control transgene expression. To overcome this shortcoming, we evaluated a cumate-inducible lentivirus (LV) construct^10^ in which *vegf-a* gene expression is regulated by a cumate repressor (CymR).

Therefore, the aim of this study was to evaluate the feasibility of using cumate-inducible LV constructs for controlling retinal transgene expression, with the ultimate goal of generating a preclinical model with controllable *vegf-a* expression with relevance to DR. For this, our initial step involved the testing of an inducible *vegf-a* expression vector within ARPE-19 cells. Subsequently, we aimed to employ this cumate-inducible *vegf-a* vector to establish a DR model in adult rats in vivo.

## Results

### Increased *vegf-a* expression in ARPE-19 cells

**Figure 1:**
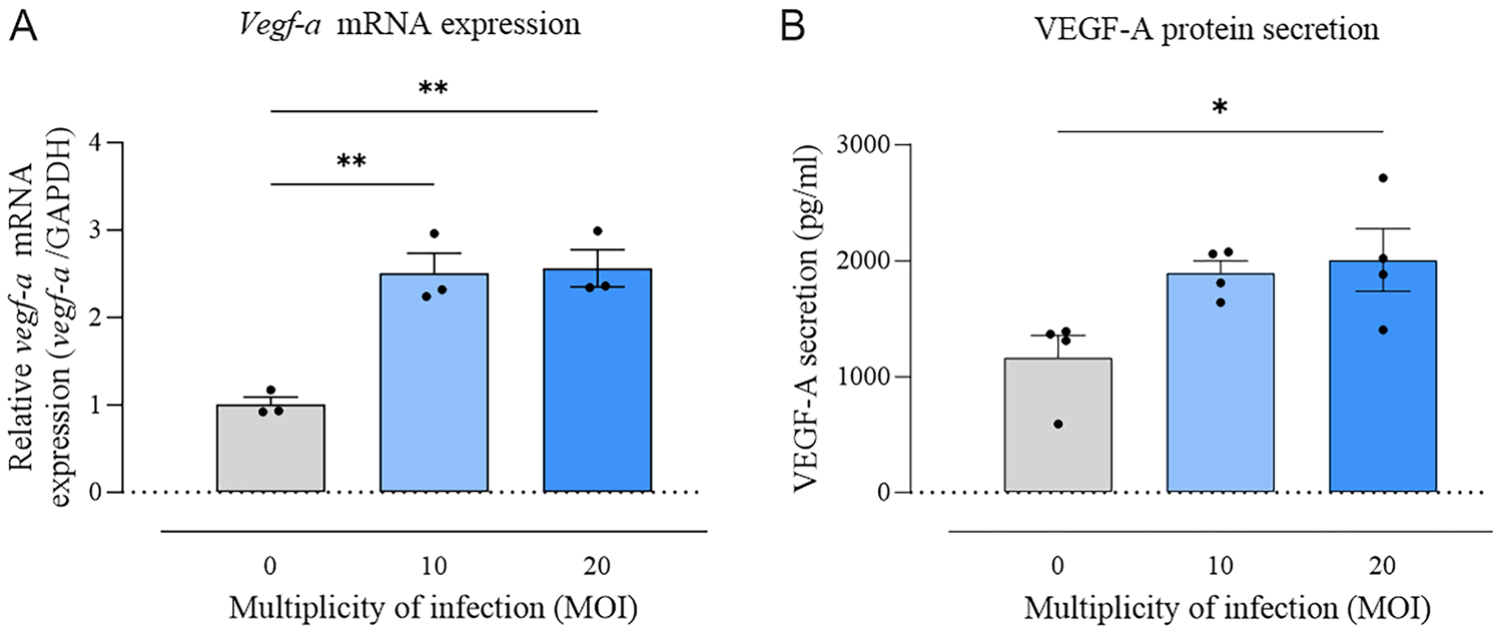
*Vegf-a* mRNA expression and VEGF-A protein secretion levels in ARPE-19 cells transduced with lentivirus at MOI 0, 10, and 20 and treated with cumate (30 µg/ml) for 48 h. (A) qPCR showed an increase of *vegf-a* mRNA expression in cells infected with LV at MOI 10 and 20 (** *p* < 0.01 and ** *p* < 0.01, respectively) compared with MOI 0. (*N* = 3/group). (B) Significantly increased VEGF-A protein secretion levels were detected by ELISA in cell supernatants transduced with lentivirus (LV) at MOI 20 (* *p* < 0.05) compared to MOI 0. (*N* = 4/group). Statistical analysis was performed using one-way ANOVA followed by Dunnett’s multiple comparisons test. Data are presented as mean ± SEM.

Lentivirus transduced ARPE-19 cell lysates were collected and *vegf-a* mRNA expression was assessed using qPCR. The results demonstrated a substantial increase in *vegf-a* mRNA levels, with approximately a 2.5-fold elevation in cells infected with LV at MOI 10 (*p* < 0.01) and nearly a threefold increase at MOI 20 (*p* < 0.01) when compared to MOI 0 (Fig. 1A). Additionally, the concentration of secreted VEGF-A in cell supernatants was quantified via ELISA. Elevated levels of secreted VEGF-A were detected in cells transduced with lentivirus at MOI 10 (1,896.60 ± 104.6 pg/ml) compared to MOI 0 (1,165.57 ± 192 pg/ml). Notably, a higher multiplicity of infection (MOI 20) resulted in a significant in-crease in VEGF-A protein secretion levels in ARPE-19 cells (2,005.60 ± 270.6 pg/ml, *p* < 0.05) compared to non-transduced cells (1,165.57 ± 192 pg/ml) (Fig. 1B).

### Effects of lentivirus-mediated expression of *vegf-a* on ARPE-19 cells

**Figure 2:**
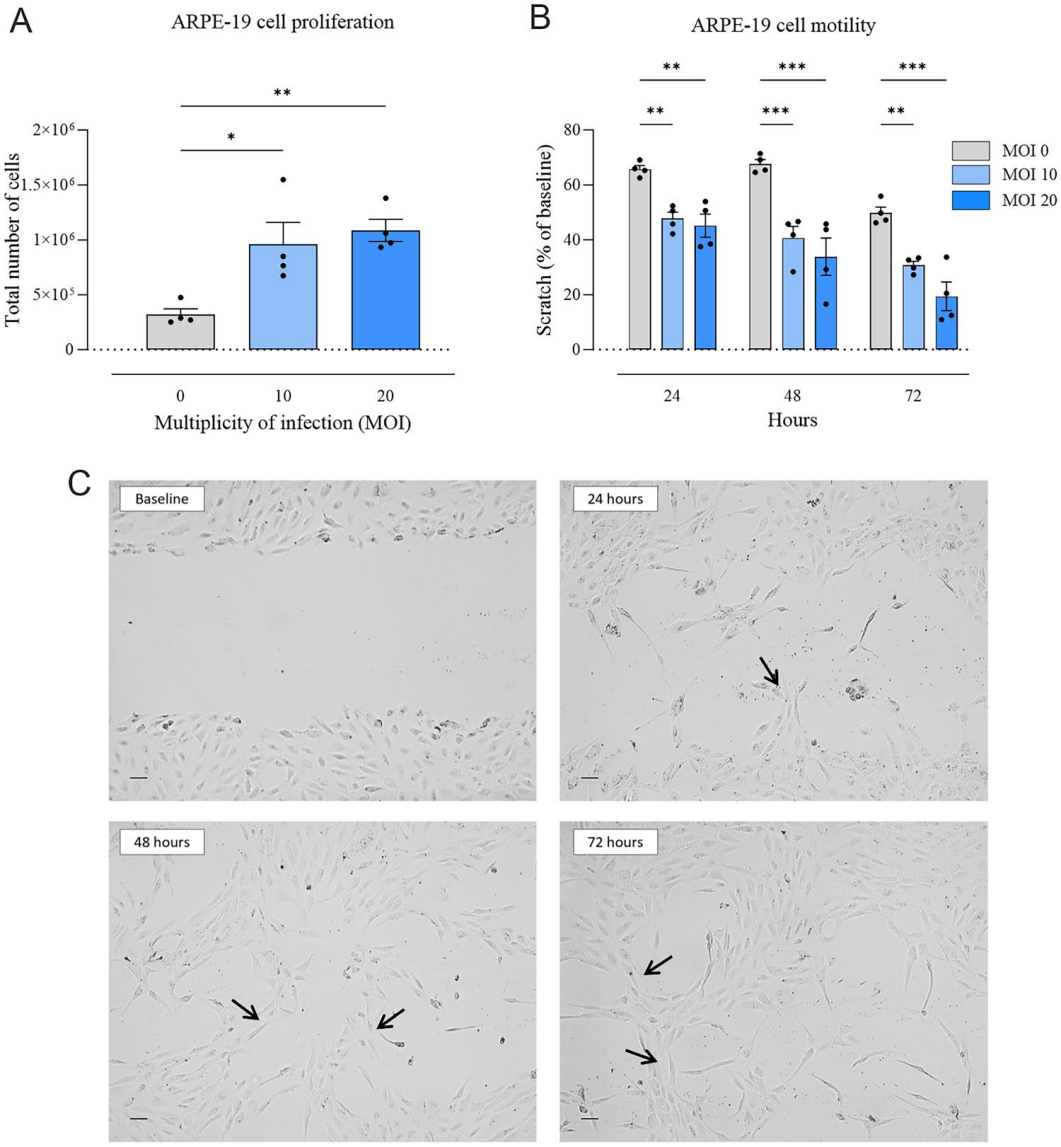
Effects of LV-mediated *vegf-a* expression on ARPE-19 cell proliferation and motility. (A) There was a significant increase in total number of ARPE-19 cells when transduced with LV at MOI 10 and 20 (* *p* < 0.05 and ** *p* < 0.01, respectively) compared with MOI 0 (*n* = 4/group). (B) There was a significant decrease in wounds suggesting an increase in cell motility when transduced with LV at MOI 10 and 20 at 24 h (** *p* < 0.01 and ** *p* < 0.01, respectively), 48 h (*** *p* < 0.001 and *** *p* < 0.001, respectively) and 72 h (** *p* < 0.01 and *** *p* < 0.001, respectively) compared with MOI 0. Statistical analysis was done by one-way and two-way ANOVA followed by Dunnett’s multiple comparisons test. Data are presented as mean ± SEM (*n* = 4). (C) Representative images of ARPE-19 cells infected with LV at MOI 20. The images were taken immediately after the scratches had been made (Baseline) and then after 24 h, 48 h and 72 h. Scratch assays showed increased motility of LV infected cells, as well as these cells forming tube-like structures, indicated by arrows. Scale bar = 100 μm.

Further, we wanted to evaluate the effects of lentivirus-mediated expression of *vegf-a* on ARPE-19 cells. The introduction of *vegf-a* through lentivirus resulted in notable enhancements in the proliferation, motility, viability, and permeability of ARPE-19 cells. For cell proliferation assessment, ARPE-19 cells were initially seeded in 12-well plates at a density of 3,000 cells per well, followed by transduction with LV and treatment with cu-mate (30 µg/ml) for 48 h. Consequently, a significant increase in the number of cells following lentivirus transduction was found at both MOI 10 (959,750 ± 200.004, *p* < 0.05) and MOI 20 (1,086,750 ± 101.268, *p* < 0.01) in comparison to MOI 0 (321,000 ± 51,828) (Fig. 2A). Furthermore, impact of *vegf-a* expression on ARPE-19 cell motility was assessed through scratch assays. A substantial reduction in scratch-wound size, indicating enhanced cell migration, was evident in cells transduced with LV at MOI 10 (40.64 ± 4.22%, *p* < 0.001) and MOI 20 (33.84 ± 6.83%, *p* < 0.001) 48 h after creating the scratch, in comparison to MOI 0 (67.67 ± 1.6%) (Fig. 2B). In addition, we observed the formation of tube-like structures by ARPE-19 cells (indicated by arrows, Fig. 2C).

**Figure 3:**
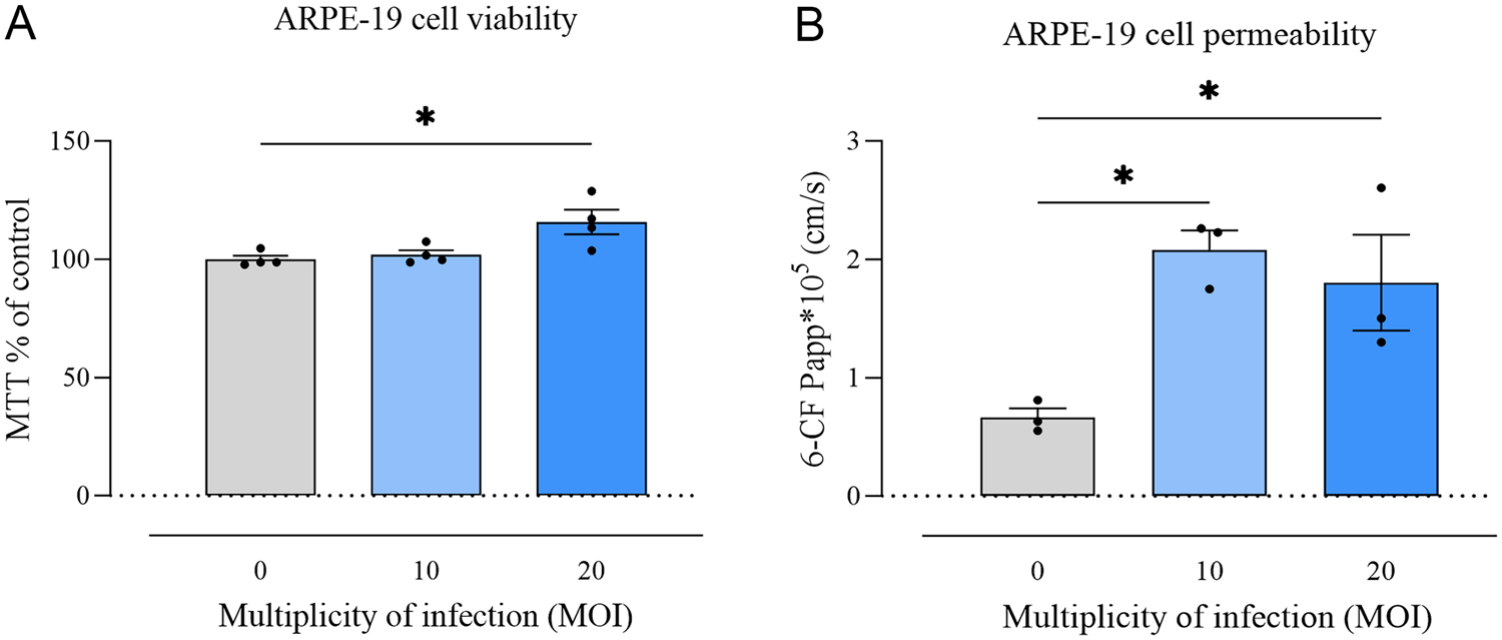
ARPE-19 cell viability and permeability after treatment with conditioned media of LV at MOI 10 and 20. (A) MTT assay showed significantly increased number of viable cells treated with conditioned media of LV at MOI 20 (* *p* < 0.05) compared with MOI 0 (*n* = 4/group). (B) Cell permeability significantly increased in cells treated with conditioned media of LV at MOI 10 and 20 (* *p* < 0.05 and * *p* < 0.05, respectively) compared with MOI 0 (*n* = 3/group). Statistical analysis was performed using one-way ANOVA followed by Dunnett’s multiple comparisons test. Data are presented as mean ± SEM.

To assess changes in cell viability, MTT assay was performed on ARPE-19 cells that were at a 70–90% confluence. Conditioned media from cells previously transduced with lentivirus and treated with cumate (30 µg/ml) for 48 h were added. After 48 h of exposure to conditioned media, the MTT assay showed significantly increased number of viable cells with conditioned media of LV at MOI 20 (115,73 ± 5.21%, *p* < 0.05), compared with MOI 0 (100 ± 1,55%) (Fig. 3A). Moreover, and MOI 20 (*p* < 0.05) compared to MOI 0 (apparent permeability coefficients Papp, 10(− 5) cm s-1 for permeability significantly increased in cells treated with conditioned media of LV at MOI 10 (*p* < 0.05).

RhoB were 2,08 ± 0.16, 1,80 ± 0.40, and 0,66 ± 0.07, respectively) (Fig. 3B). Our in vitro data are consistent with the anticipated effects of *vegf-a* overexpression in ARPE-19 cells.

### Toxic effect of cumate IVT injections on retina of wistar rats

**Figure 4:**
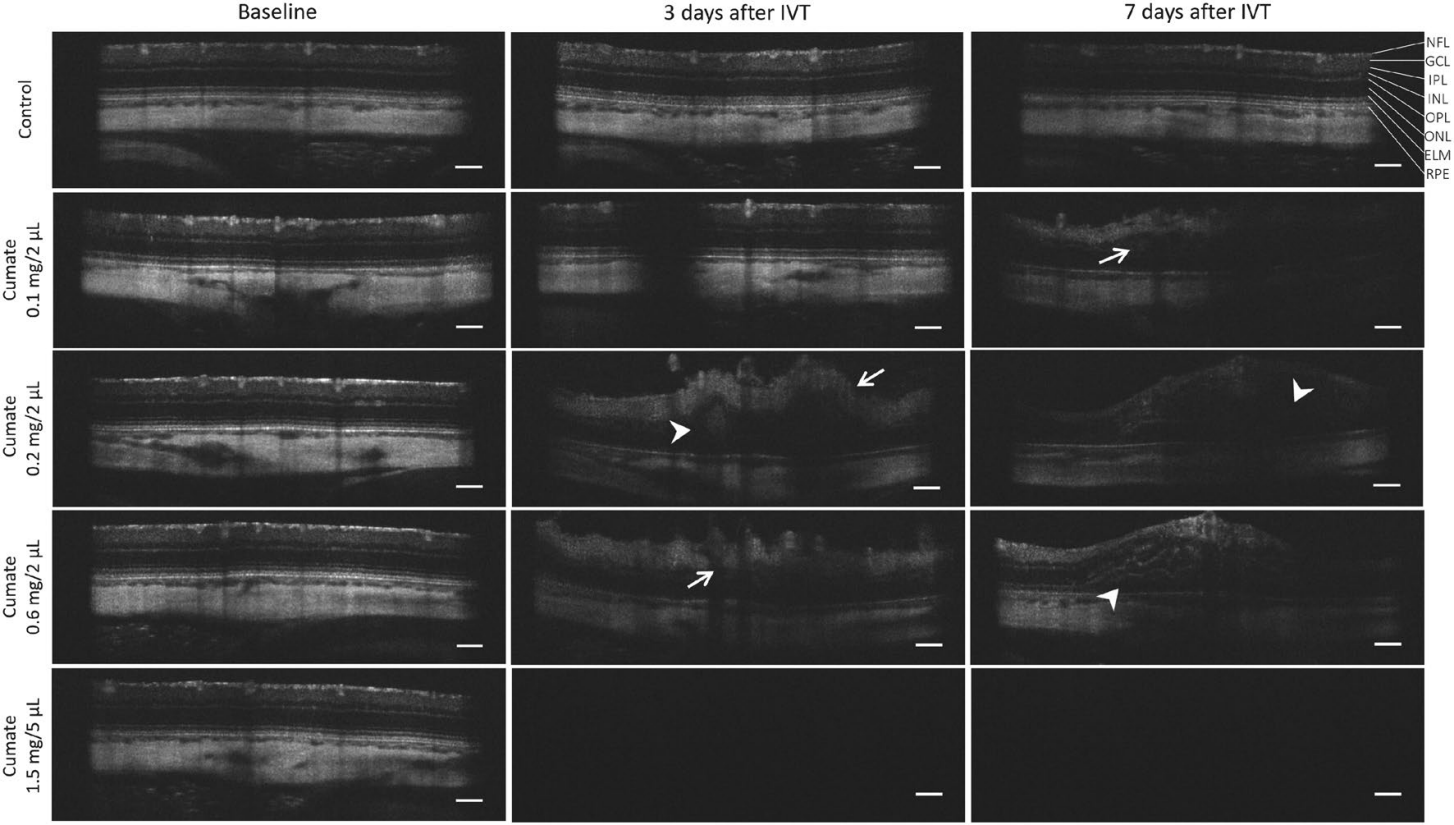
IVT injection of cumate induced retinal degeneration in rat eyes. Cumate (administered at doses of 0.1 mg/2 µL; 0.2 mg/2 µL; 0.6 mg/2 µL, and 1.5 mg/5 µL) resulted in pathological retinal layering (arrows), accumulation of fluid within retinal layers, retinal hemorrhages (arrowheads), and the presence of vitreous opacities (bottom row) which completely prevented SD-OCT imaging on Day 3 and Day 7 post-IVT injection. NFL = nerve fiber layer; GCL = ganglion cell layer; IPL = inner plexiform layer; INL = inner nuclear layer; OPL = outer plexiform layer; ONL = outer nuclear layer; ELM = external limiting membrane; RPE = retinal pigmented epithelium. Scale bar = 200 µm.

Our next objective was to assess the feasibility of utilizing cumate-inducible lentiviral (LV) constructs to establish inducible gene expression in an in vivo setting. Given the lack of in vivo data on cumate, we conducted an evaluation to determine cumate's tolerability introduced via intravitreal injections. Following IVT injection of cumate (administered at doses 1 mg/2 µL, 0.2 mg/2 µL, 0.6 mg/2 µL, or 1.5 mg/5 µL), the rat eyes exhibited immediate cloudiness, indicating adverse reactions to cumate. SD-OCT scans revealed noticeable changes in the retinal layers (indicated by arrows in Fig. 4), as well as the accumulation of fluid within retinal layers and retinal hemorrhage (indicated by arrowheads in Fig. 4). Additionally, in majority of SD-OCT scans from rats receiving the highest dose of cu-mate (1.5 mg/5 µL), the observations were obscured due to cumate-induced vitreous opacities that cast shadows on the retina (bottom row of Fig. 4).

**Figure 5:**
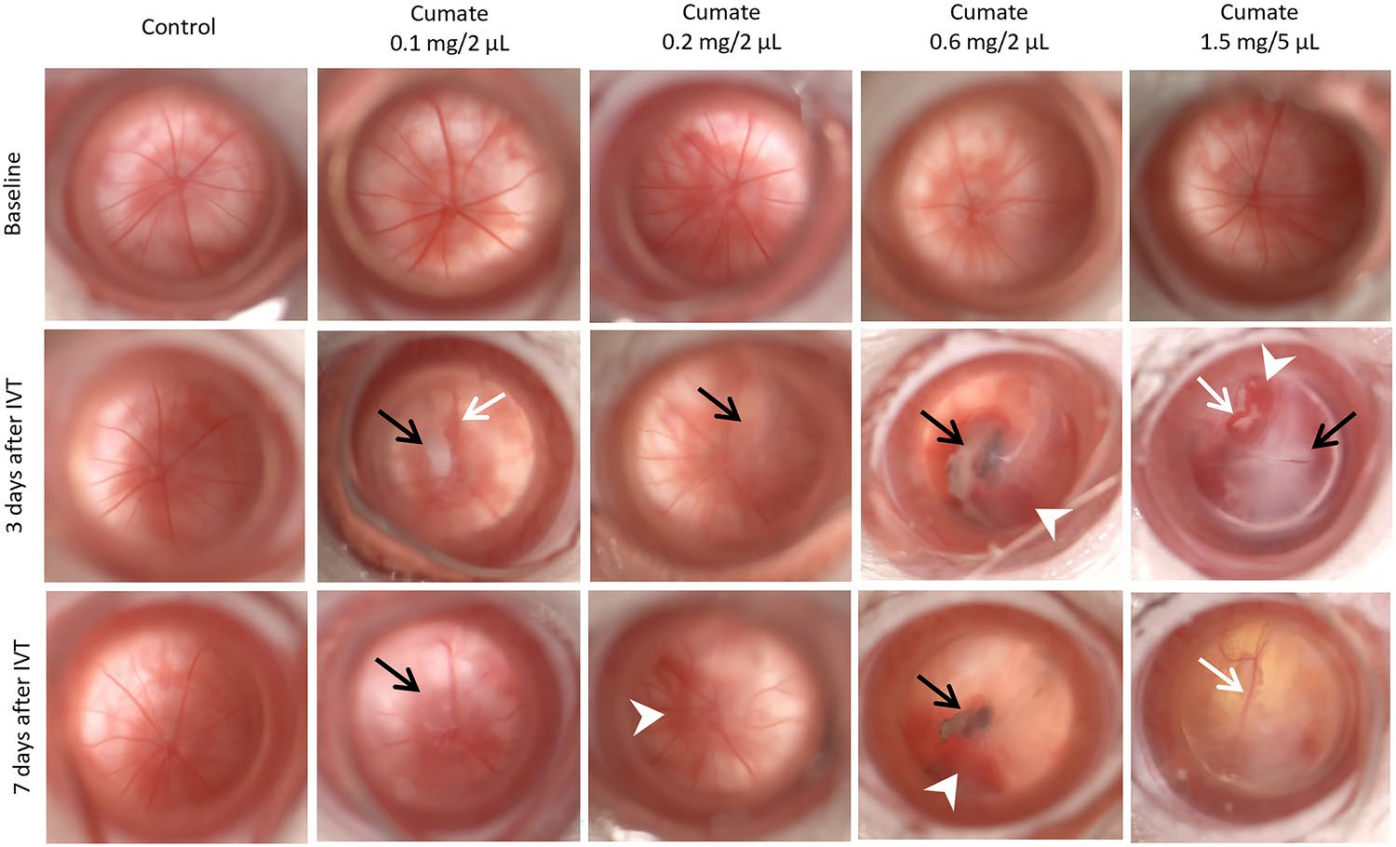
Administration of cumate (at doses 0.1 mg/2 µL; 0.2 mg/2 µL; 0.6 mg/2 µL, and 1.5 mg/5 µL) resulted in vascular pathologies in rat eyes. Enlarged or constricted blood vessels were noticed during fundus observations (white arrows) 3- and 7-days post-IVT-administration. Severe retinal hemorrhages were detected 3 days post-IVT which persisted until Day 7 (white arrowheads). Vitreous opacities were observed at 3 days and at 7 days after cumate administration (black arrows).

Further, ophthalmic examinations revealed vascular abnormalities in rat eyes, both 3- and 7-days post-administration of cumate (at doses 0.1 mg/2 µL; 0.2 mg/2 µL; 0.6 mg/2 µL, or 1.5 mg/5 µL). Intravitreal injections of cumate induced distinct alterations in retinal blood vessels, characterized by either vessel enlargement or constriction (highlighted by white arrows in Fig. 5). The fundus images provided further evidence of retinal hemorrhages, which were consistent with observations in SD-OCT scans 3- and 7-days after cumate administration (indicated by arrowheads in Fig. 5). Additionally, ophthalmic examinations identified vitreous opacities, likely caused by the presence of cumate suspended within the vitreous (denoted by black arrows in Fig. 5).

**Figure 6:**
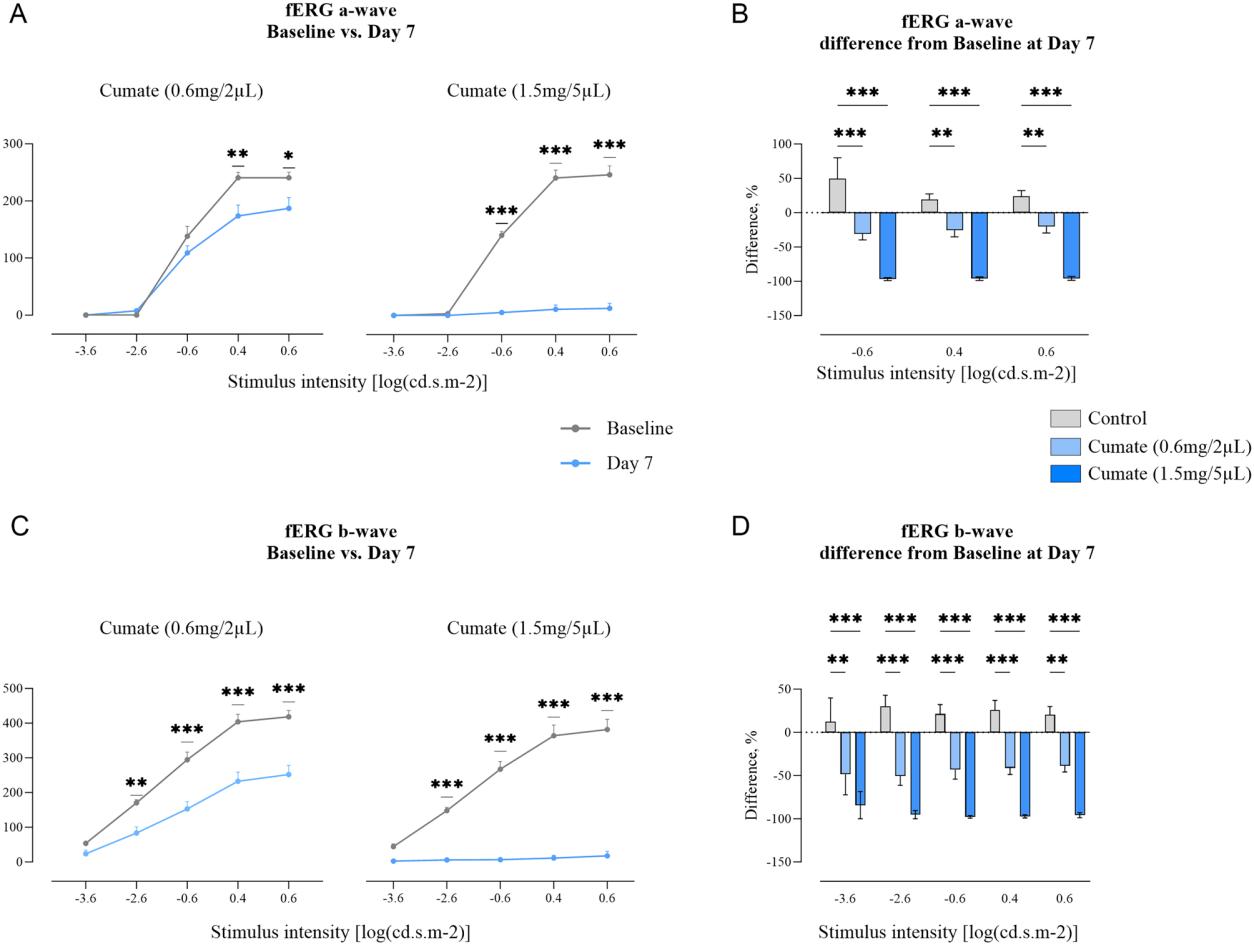
Cumate IVT injections (at doses 0.6 mg/2 µL and 1.5 mg/5 µL) led to decreased retinal activity. Retinal function analyzed by fERG at stimulus intensities -3.6, -2.6, -0.6, 0.4, 0.6 [log (cd.s.m-2)]. (**A**) a-wave amplitudes at Baseline and Day 7, and (**B**) difference from Baseline at Day 7 in different treatment groups. (**C**) b-wave amplitudes at baseline and Day 7, and (**D**) difference from Baseline at Day 7 in different treatment groups. Statistical analysis was done by two-way ANOVA followed by Šídák's or Dunnett's multiple comparisons test * *p* < 0.05, ** *p* < 0.01, *** *p* < 0.001. Data are presented as mean ± SEM, *n* = 8–12 eyes/group.

Flash electroretinography (fERG) was performed to determine the impact of cumate IVT injections on rat retinal function. Seven days after cumate administration (0.6 mg/2 µL), a-wave amplitude decreased by 22%, and b-wave amplitude by 39% (0.6 [log (cd.s.m-2)] stimulus intensity, *p* < 0.05 and *p* < 0.001, respectively; Fig. 6). Higher cumate concentrations (1.5 mg/5 µL) resulted in a more substantial reduction, with a 94% decline in a-wave amplitude and a 95% decrease in b-wave amplitude by Day 7 (0.6 [log (cd.s.m-2)] stimulus intensity, *p* < 0.001 and *p* < 0.001, respectively; Fig. 6). However, it is important to note that these a- and b-wave amplitude decreases might be due to cumate-induced toxicity to the retina, vitreous opacities, or a combination of both factors (all fERG a-wave and b-wave absolute values can be found in Supplementary Table S1).

**Figure 7:**
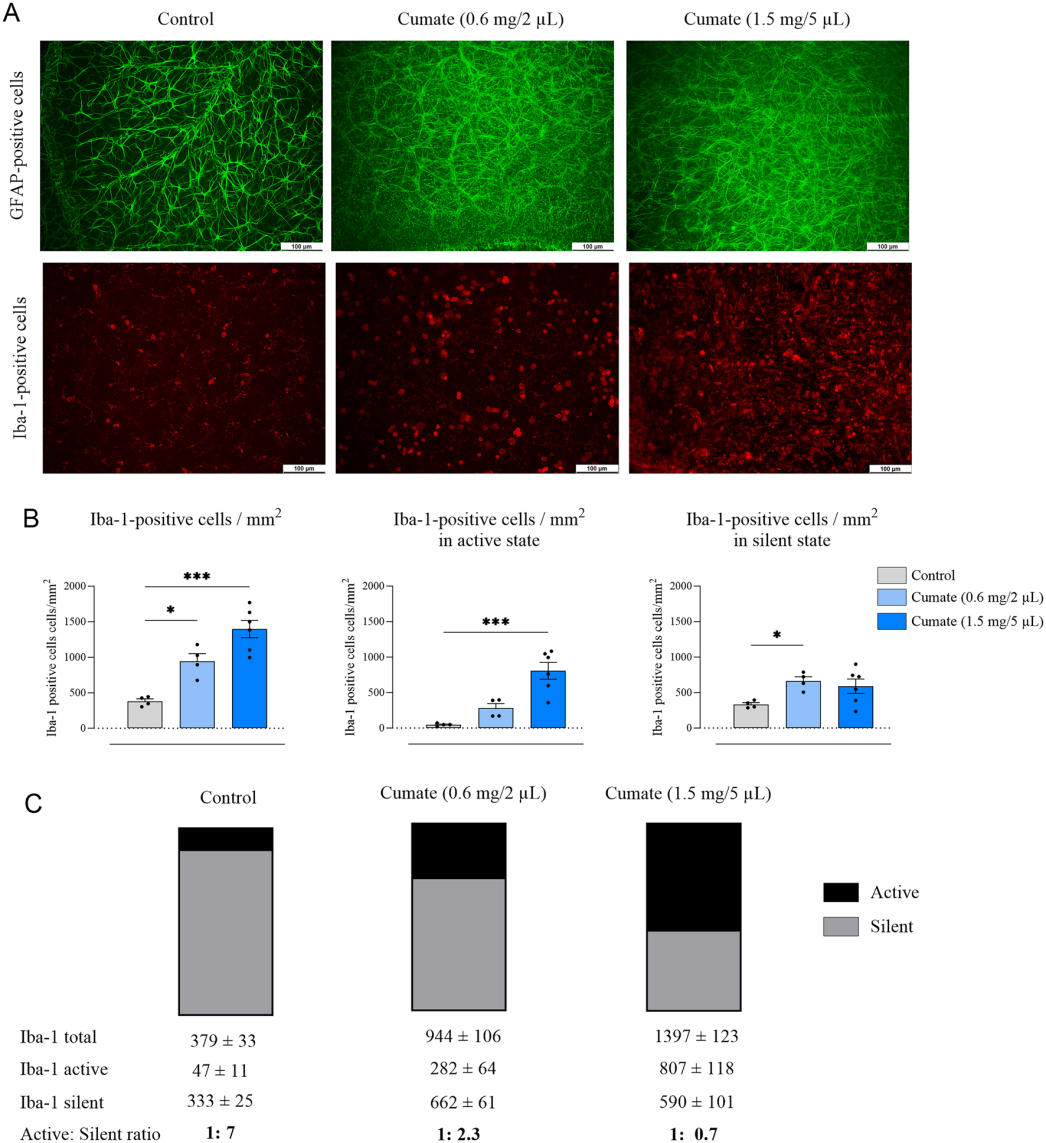
Assessment of immune cell activation in cumate-injected (0.6 mg/2 µL or 1.5 mg/5 µL) eyes. (**A**) Representative images of retinal flat-mounts from cumate-treated eyes stained with GFAP and Iba-1. (**B**) The count of Iba-1^+^ cell numbers per 1 mm^2^. Significant increases in total, active and silent Iba-1^+^ cell numbers were observed in eyes treated with cumate (one-way ANOVA followed by Dunnet’s multiple comparisons test *, *p* < 0.05, ***, *p* < 0.001). (**C**) Ratio between silent and active Iba-1^+^ cells across all treatment groups. Data are presented as mean ± SEM, *n* = 4–6 eyes/group.

Next, retinas exposed to cumate were immunostained with Iba-1 and GFAP antibodies to assess the activation of microglia and the presence of reactive gliosis, respectively. Seven days after IVT injection of cumate (0.6 mg/2 µL or 1.5 mg/5 µL), retinas were largely disorganized, and GFAP^+^ astrocytes were significantly increased across the full retina (top panel in Fig. 7A). Moreover, significantly increased number of Iba-1^+^ microglia were detected within the retinal tissue following IVT injection of cumate at doses of 0.6 mg/2 µL (944 ± 106 cells/mm^2^, *p* < 0.05) and 1.5 mg/5 µL (1397 ± 123 cells/mm^2^, *p* < 0.001) in comparison to control rat retinas (379 ± 33 cells/mm^2^, Fig. 7B). Moreover, an increased number of activated Iba-1 + microglia was found in the highest dose of cumate group (1.5 mg/5 µL) (807 ± 118 cells/mm^2^ compared to 47 ± 11 cells/mm^2^ in control group, *p* < 0.001). Cumate (at a dose of 1.5 mg/5 µL) resulted in markedly reduced active Iba-1 to silent Iba-1 ratio (1: 0.7) in comparison to control rats (1: 7), indicating that higher proportion of Iba-1^+^ cells were in an active state than silent (Fig. 7C).

**Figure 8:**
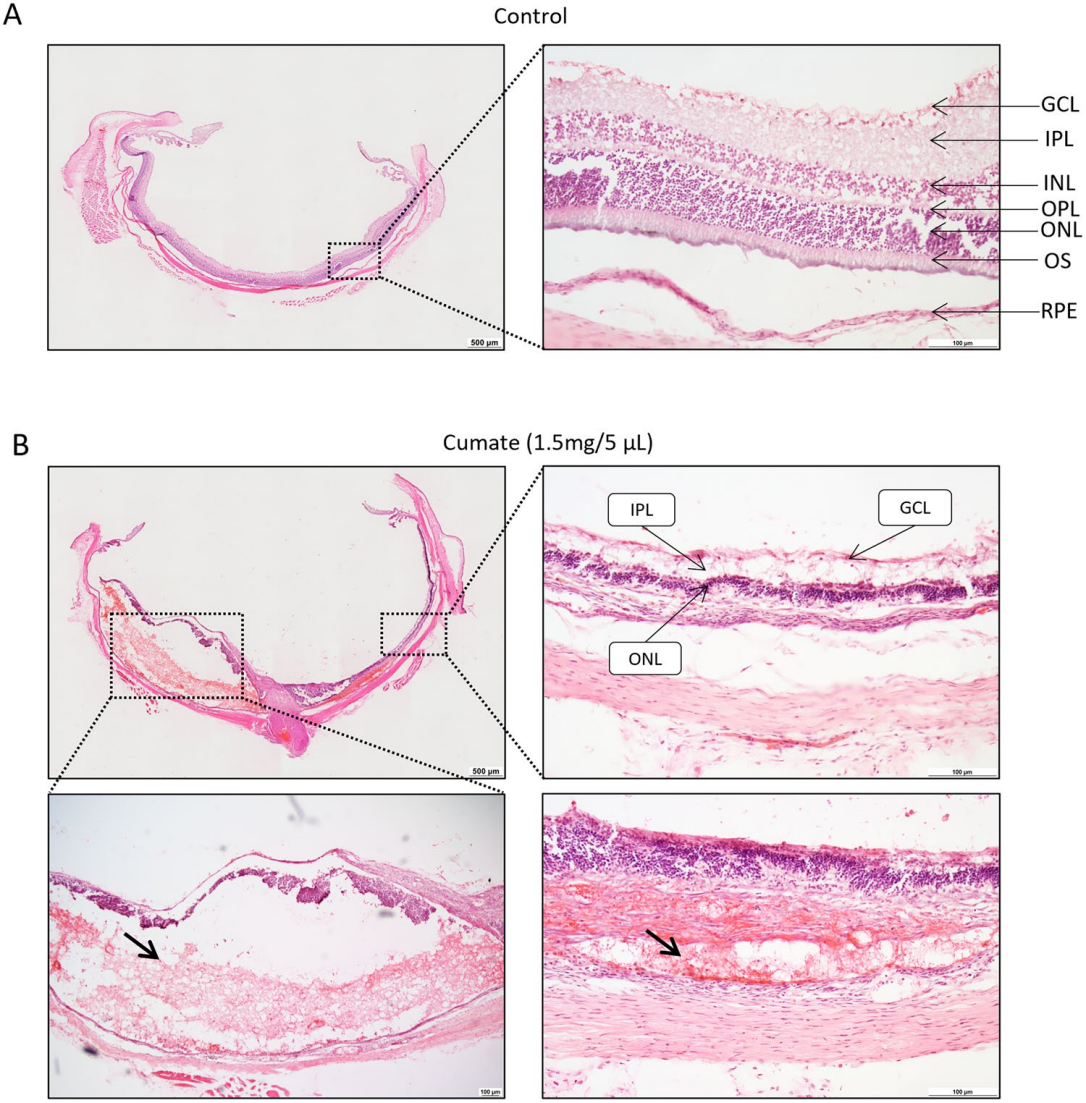
Hematoxylin and eosin (H&E) staining of eye cup cross-sections of (**A**) control and (**B**) cumate (1.5 mg/5 µL)-injected eyes. Retinal haemorrhages (black arrows), irregular retinal layers and retinal degeneration (e.g., IPL, ONL, and GCL) were observed seven days after cumate administration. Scale bars: 100 μm and 500 μm. GCL = ganglion cell layer; IPL = inner plexiform layer; INL = inner nuclear layer; OPL = outer plexiform layer; ONL = outer nuclear layer; OS = outer segments of photoreceptor cells; RPE = retinal pigmented epithelium.

To verify cumate-induced toxicity histologically, eye cup cross-sections of injected eyes were evaluated (Fig. 8). Hematoxylin and eosin (H&E) staining revealed normal architecture of the retina in control eye (Fig. 8A) whilst distinct indications of retinal degeneration were evident at seven days after IVT injection (Fig. 8B). Notably, retinal hemorrhages (indicated by black arrows in Fig. 8B), as well as anomalous morphology of various retinal layers (e.g., ganglion cell layer (GCL), inner plexiform layer (IPL), and outer nuclear layers (ONL), Fig. 8B) were evident in the cumate-injected eyes. These findings were in alignment with our prior observations from SD-OCT scans and ophthalmic examinations. Collectively, all four administered doses of cumate (0.1 mg/2 µL, 0.2 mg/2 µL, 0.6 mg/2 µL, and 1.5 mg/5 µL) were associated with varying degrees of retinal degeneration when compared to the control group that received a 5 µL of saline injection.

## Discussion

The aim of our study was to evaluate the feasibility of using cumate-inducible LV constructs for controlling retinal transgene expression, with the ultimate goal to generate a DR preclinical model with controllable *vegf-a* expression. Transduction of ARPE-19 cells with cumate-inducible LV led to elevated *vegf-a* mRNA and VEGF-A protein secretion levels. Transduced cells displayed enhanced cell proliferation, viability, permeability, and migration in tube-like structures. However, IVT cumate injections led to evident retinal toxicity, manifesting as retinal layer abnormalities, hemorrhages, vitreous opacities, and significant reductions in a- and b-wave amplitudes, along with increased microglial activation and reactive gliosis.

Studies demonstrate that cumate-inducible gene expression vectors can be used as a system for regulated expression in mammalian^10^, human embryonic kidneys^11^, and other cell lines^12–14^. However, to our knowledge, cumate-inducible gene expression vectors were never reported to be used for ophthalmic discoveries in in vivo systems. The anticipated advantage over alternative constructs, including adeno-associated virus (AAV), lies in the unique capability of the cumate-inducible system to provide titratable and finely tuned control over gene expression. Other inducible systems, such as the widely used Tetracycline (Tet) inducible expression system, also offer opportunities for regulated gene expression, but come with specific challenges such as leaky expression in the absence of the inducer^15^. By leveraging the cumate-inducible system, our study aimed to address this need for precise control and explore the potential for novel insights into the molecular mechanisms underlying VEGF-A-related pathologies, ultimately contributing to the development of innovative animal model for DR.

Our initial experimental step involved the transduction of ARPE-19 cells with cumate-inducible LV expressing *vegf-a* at varying multiplicity of infection (MOI) levels, including MOI 0, 10, and 20. VEGF-A is a well-known growth factor that is highly upregulated in DR^16^, serves as a pivotal element in the context of our investigation. In our study, qPCR showed an increase of *vegf-a* mRNA expression within cells infected with LV at MOI 10 and 20, compared to MOI 0. Furthermore, significantly increased VEGF-A protein secretion levels into the cell supernatant were detected by ELISA upon transduction with LV at MOI 20 as compared to MOI 0. Additionally, we observed the effects of lentivirus-mediated expression of *vegf-a* on ARPE-19 cells. In pathologic conditions of DR, VEGF-A is known to stimulate excessive proliferation and migration of retinal cells^17^. Our investigation revealed a significant rise in the total number of ARPE-19 cells, indicating an increase in cell proliferation after transduction with lentivirus at MOI 10 and 20. Moreover, we examined the influence of *vegf-a* expression on ARPE-19 cell motility through scratch-wound assays. This analysis showed a noteworthy reduction in the scratch-wound size, signifying an increase in the migratory capacity of ARPE-19 cells upon transduction with lentivirus at MOI 10 and MOI 20. Elevated levels of VEGF-A are associated with the promotion of tube formation in retinal endothelial cells^18^, which plays a crucial role in the development of new blood vessels, particularly in the context of diabetic retinopathy^4^. In our study we observed LV-transduced ARPE-19 cells forming tube-like structures, further suggesting the presence and activity of VEGF-A.

Studies have established that VEGF-A plays a pivotal role in enhancing the survival of retinal pigment epithelium (RPE) cells^19^. In our study the MTT assay revealed a substantial increase in the number of viable cells when exposed to conditioned media of lentivirus at MOI 20, as opposed to MOI 0. Furthermore, prior research has indicated that the activation of VEGF receptors located on the apical surface of RPE cells is responsible for the VEGF-induced elevation in cell permeability^20^. Our study findings corroborate these observations, as we found a significant increase in permeability among ARPE-19 cells transduced with conditioned media of LV at both MOI 10 and MOI 20. Taken together, cumate-inducible lentivirus promotes the expression of *vegf-a* and secretion of VEGF-A protein in ARPE-19 cells, resulting in enhanced cell proliferation, viability, permeability, and migration in tube-like structures. Our data demonstrate that cumate-inducible LV is a flexible tool to study the function of VEGF-A in vitro, and potentially in vivo.

Hence, next, we aimed to evaluate cumate-inducible lentivirus in vivo. With the SparQ™ Cumate Switch System, inducible *vegf-a* gene expression is reached through the binding of cumate to the cumate repressor (CymR), and expression levels of *vegf-a* are tightly controlled with increasing cumate concentration. Therefore, in this study we wanted to evaluate the tolerability of cumate in rat eyes, where adult Wistar rats received single bilateral IVT injection of cumate at varying doses: 1 mg/2 µL; 0.2 mg/2 µL; 0.6 mg/2 µL, or 1.5 mg/5 µL. However, cumate was not tolerated and resulted in significant retinal toxicity in rat eyes. Immediately after cumate administration, rat eyes appeared cloudy, signaling adverse reactions to cumate. SD-OCT scans revealed notable morphological changes of retinal layers, fluid accumulation, and the presence of retinal hemorrhages. Moreover, cumate caused vitreous opacities that cast shadows on the retina, therefore most of the SD-OCT scans performed on rats which received highest dose of cumate (1.5 mg/5 µL) appeared completely negative. Ophthalmic examinations provided further confirmation of retinal pathologies induced by cumate administration, including the alterations in the size of retinal blood vessels, hemorrhages, and the presence of vitreous opacities, likely attributed to the presence of cumate suspended within the vitreous body. To further assess the impact of cumate IVT injections, retinal function was evaluated using fERG. By day seven following IVT injections, there was a notable reduction in both a-wave and b-wave amplitudes. However, this decline in a- and b-wave amplitudes may be attributed to the potential toxicity of cumate to the retina, the presence of vitreous opacities, or a combination of these two factors.

In addition to this, immunohistochemistry analysis of retinal flat-mounts revealed enhanced immune cell activation in cumate-treated eyes. Following IVT injection of cumate, retinas were largely disorganized, and there was a notable increase in GFAP^+^ astrocytes throughout the entire retina, indicative of heightened reactive gliosis. Moreover, our findings demonstrated a substantial increase in the total number of Iba-1^+^ cells within the retina, signifying microglia activation. Notably, cumate resulted in significantly lower active Iba-1: silent Iba-1 ratio compared to control rats, showing that more microglia cells were in active state than silent. Furthermore, eye cup cross-sections of cumate treated eyes were H&E stained and evaluated. The presence of retinal degeneration was found and verified our observations from SD-OCT scans and ophthalmic examinations. The assessment revealed occurrence of retinal hemorrhages and the presence of abnormal morphology of retinal layers following cumate administration. Collectively, these findings from our animal experiments provide strong evidence that cumate exerts a toxic effect and induces retinal degeneration in Wistar rats.

Given the observed toxicity of cumate following intravitreal injection in this study, future research may investigate the pharmacokinetic properties of cumate in ocular subtissues and evaluate the feasibility of using lower concentrations to initiate *vegf-a* expression. However, it is likely that multiple low-dose intravitreal injections may be required to achieve relevant tissue levels of cumate. While recent work has elegantly demonstrated that oral administration of cumate is safe and feasible,^21^ it remains unclear whether cumate can effectively cross the retinal-blood barrier and/or blood–brain barrier, which is crucial for delivering cumate to the eye and brain.

In conclusion, our study demonstrates that the cumate-inducible *vegf-a* expression system offers a valuable tool for investigating the dose-dependent effects of VEGF-A on ARPE-19 cells. Generation of stably expressing cell line could offer potential applications for VEGF-related in vitro studies. However, the observed retinal damage induced by cumate in rat eyes precludes its use via intravitreal injections. This limitation prevents us from developing an easy-to-use inducible DR rat model and from gaining insights into the pathology of diabetic retinopathy using a cumate-inducible lentivirus expressing *vegf-a*.

## Methods

### Cell line and cell culture

Retinal pigment epithelial cells, ARPE-19 (CRL-2302TM, American Tissue Type Col-lection, ATTC, Manassas, VA, USA), were cultured in a 1:1 mixture of Dulbecco's modified Eagles medium (DMEM) and Ham's F12 medium (F12 medium) (containing 1.2 g/L sodium bicarbonate, 2.5 mM L-glutamine, 15 mM HEPES and 0.5 mM sodium pyruvate) and fetal bovine serum to a final concentration of 10% and cultured at 37 °C in an atmosphere of 5% CO^2^/95% humidity. Cell cultures were supplemented with penicillin/streptomycin to a final concentration of 100 U/ml and were used for experiments be-tween passages 7–10.

ARPE-19 were maintained in T75 tissue culture flasks (TPP Techno Plastic Products AG, Trasadingen, Switzerland) and seeded into clear, flat bottom 6, 12, or 96 well plates (TPP®; Midwest Scientific) at a density of 6900 cells/well to 200,000 cells/well. For permeability experiments, cells were seeded in transwell inserts (150,000 cells/well; 12 mm Transwell® with 0.4 µm Pore Polyester Membrane Inserts (Corning; Corning, NY, USA]).

### ARPE-19 cell transduction with cumate-inducible lentivirus (LV)

ARPE-19 cells were incubated at 37 °C with 5% CO_2_ overnight so that the cells would be 10–30% confluent at the time of infection. TransDux™ (System Bioscience, Palo Alto, CA, USA) was added to cell medium to a final concentration of 1X, for efficient transduction of cells. Immediately after, ARPE-19 cells were transduced with lentivirus (LV) expressing a SparQ™ all-in-one cumate-inducible plasmids (QM800A-VEGFA_165A_, 1.14 × 109 ifus/ml [System Biosciences]) expressing *vegf-a* 165 isoform (VEGF-A_165_) at a multiplicity of infection (MOI) of 0, 10, and 20. Media was changed 72 h post-transduction and cumate (30 μg/ml [System Biosciences]) was added to induce *vegf-a* expression, and cells were incubated for a further 48 h.

### RNA isolation and real-time quantitative PCR (RT-qPCR)

Total RNA from transduced ARPE-19 cell lysates were isolated using the commercially available Total RNA Purification Plus Kit (Norgen, Biotek, Ontario, Canada), according to the manufacturer's protocol, and quantified using a NanoDrop spectrophotometer (ThermoFisher Scientific Inc, Wilmington, DE, USA). An equal amount of RNA (92 ng) from each sample was used to synthesize single-stranded complementary DNA (cDNA) via reverse transcription (High-Capacity RNA-to-cDNA Kit, Thermo Fisher Scientific). The resulting cDNA was then subjected to real-time quantitative PCR (RT-qPCR) (AriaMx Real-time PCR System) for *vegf-a* (Hs00900055_m1) expression. Human GAPDH (GAPDH, 4326317E) was used as the endogenous control for gene expression normalization. All qPCR probe sets, Taqman Gene Expression Assay kit and Taqman fast advanced Master Mix were acquired from Applied Biosystems (Thermo Fisher Scientific). The assays were conducted in triplicates for each sample. (*N* = 3/group).

### Enzyme-linked immunosorbent assay (ELISA)

Transduced ARPE-19 cell supernatant samples were prepared, and VEGF-A protein levels were determined by an investigator masked to the treatment conditions, using a commercial VEGF-A ELISA kit (R&D Systems, MN, USA). The assay procedure was per-formed according to the manufacturer’s instructions, with the modification of a 1:5 dilution of the samples in RD5K diluent. To determine VEGF-A concentrations, a range of standards (31.3, 62.5, 125, 250,500, and 1000 pg/ml) were used. Absorbance was measured at 450 nm using a Cytatation 5 microplate reader (BioTek Instruments Inc., Winooski, VT, USA), with subtraction of the absorbance of blank wells at 540 nm as a reference. (*N* = 4/group).

### Scratch assays

The effects of LV-mediated VEGF-A expression on ARPE-19 cell motility were assessed through scratch assays. Cells transduced with LV at MOI 0, 10, and 20 were incubated for 72 h after a scratch was created using a 10 μl pipette tip. These cells were cultured in a serum-free F12/DMEM medium at 5% CO2/95%, 37 °C, in the presence of cumate (30 μg/ml). Images were taken immediately after the wound was made, then at 24, 48, and 72 h. Initial wound dimensions and wound healing were evaluated using Image J software (v1.46r, National Institute of Health, USA) by an investigator masked to the treatment conditions. (*N* = 4/group).

### MTT assay

ARPE-19 cell viability was evaluated by 3-(4,5-dimethylthiazol-2-yl)-2,5-diphenyltetrazolium bromide (MTT) uptake assay. Conditioned media of LV-*vegf-a*-treated cells at MOI 0, 10, and 20 was added to ARPE-19 cells seeded in 96-well plate. In 48 h, media was aspirated from the cells and replaced with 100 μl of 1.2 mM MTT in Hanks' Balanced Salt solution (HBSS) with calcium and magnesium (Lonza, Walkersville, MD, USA) supplemented with 10 mM 4-(2-Hydroxyethyl) pi-perazine-1-ethanesulfonic acid (HEPES; Sigma Aldrich, St Louis, USA). Plates then were incubated at 37 °C for 2 h. Media was aspirated, and cells were lysed with 100 μl dimethylsulfoxide (DMSO) and gentle shaken, to lyse the cells. Absorbance was measured at 570 nm using a Cytatation 5 microplate reader (BioTek Instruments Inc.). Absorbance values were corrected for background and normalized to the control condition (MOI 0). (*N* = 4/group).

### Cell permeability

To examine possible changes in ARPE-19 cell permeability in the presence of conditioned media from LV-*vegf-a*-treated cells, cells were grown to 70–90% confluency in transwell inserts. Cells were then treated with conditioned media of LV-*vegf-a*-treated cells at MOI 0, 10, and 20 for 48 h. To assess permeability, the apical to basolateral movement of the low-permeability dye, 6-carboxyfluorescein (6-CF; ThermoFisher Scientific) was quantified, using an excitation 490 nm/emission 520 nm, and a microplate reader (Cytatation 5; BioTek Instruments Inc.). (*N* = 3/group).

### Animal experiments

All animals were approved and monitored by the Animal Welfare Ethical Board of Lithuania (Experimentica UAB animal license number G2-136). Animals were also treat-ed in accordance with the ARVO Statement for the Use of Animals in Ophthalmic and Vision Research and the EC Directive 2010/63/EU for animal experiments. No power calculations were performed to determine group sizes but were based on previous experiments in similar designs. Animals were randomly assigned to treatment groups and experimenters were masked to the treatment conditions. The study is reported in accordance with ARRIVE guidelines.

For cumate tolerability experiments, seven-weeks old male Wistar rats (Janvier, France) (weight, 220–250 g) were housed at a constant temperature (22 ± 1 °C) in a light-controlled environment (lights on from 7 am to 7 pm) with ad libitum access to food and water. For all the procedures, animals were anesthetized with ketamine (45 mg/kg; Ketamidor, Richter Pharma, Austria) and medetomidine (0.6 mg/kg; Sedator, Eurovet animal health, Spain) mixture. Anesthesia was reversed by the α2-antagonist for medetomidine, atipamezole (2.5 mg/kg; Atipam; Eurovet Animal Health, The Netherlands). At the end of the study rats were euthanized by cervical dislocation under non-recovery deep anaesthesia, and eyes were enucleated and prepared for immunohistochemical staining (as described below). (*N* = 4–12 eyes/group).

### Intravitreal injections

Water-soluble cumate solution (10,000 x, System Bioscience, Palo Alto, CA, US) was administered by a single bilateral intravitreal injection (IVT) into both eyes of randomly selected rats. Four distinct doses of cumate were administrated: 0.1 mg/2 µL (6 eyes), 0.2 mg/2 µL (4 eyes), 0.6 mg/2 µL (10 eyes), and 1.5 mg/5 µL (12 eyes). As for control group, 5 µL of saline was administrated. These dosed of cumate were selected based on our in vitro studies and the prior literature indicating the efficacy of cumate at these doses for binding to the cumate repressor and inducing VEGF-A expression. For dose selecton, we took also into account cumate half-life, the diluton of cumate into the rat vitreous and the need for biodistribution from the vitreous into the retina. The injections were carried out on Day 0 of the study using a 5 µl glass micro syringe (Hamilton Bonaduz AG, Bonaduz, Switzerland).

### Spectral domain optical coherence tomography (SD-OCT)

Cumate tolerability was assessed using spectral domain optical coherence tomography (SD-OCT) imaging obtained with an image-guided OCT system (Envisu R2210, Bioptigen Inc./Leica Microsystems). SD-OCT scans were conducted on Day 0 prior to the IVT injections, Day 3, and the final experimental day (Day 7). The scanned area covered a 1.8 × 1.8 mm of the retina centered around the optic nerve. Each scan was composed of 100 B-scans, each one composed of 1000 A-scans.

### Ophthalmic examination (OE)

Ophthalmic examinations (OE) were performed using slit lamp (SL-9900, C.S.O srl, Italy). Prior to the examinations, a 10 mg/ml tropicamide (Polfa S.A, Poland) solution was administered onto the cornea to dilute pupils. Rats were then placed under the slit lamp, and both eyes were examined, with images captured at both cornea and fundus levels. Ophthalmic examinations were conducted on Day 0 before the intravitreal injections of cumate and then on days 3 and 7 post-injection.

### Flash electroretinography (fERG)

All flash electroretinography (fERG) measurements were conducted with animals dark-adapted overnight in their home cages, with ad libitum access to food and water. Rats were anesthetized as described above and their pupils were dilated using a 10 mg/ml tropicamide solution (Polfa S.A, Poland). Oftagel (Santen) at a concentration of 2.5 mg/g was applied to keep the eyes moist during recording. Recordings were performed on both eyes under complete darkness. This was achieved using an AC/DC differential amplifier (A-M SYSTEMS, USA, MODEL 3000) and a data acquisition interface (C.E.D., United Kingdom, Power1401). Light flash stimuli (3ms in duration) of five different intensities were used, in ascending order: − 3,6; − 2,6; − 0,6; 0,4 and 0,6 [log (cd.s.m-2)], with an inter-stimulus interval of 5s for the two lowest intensities and 10s for the rest. The response to each intensity were recorded for ten consecutive times, averaged, and further used to manually identify the amplitudes (µV) for both a- and b-waves. fERG measurements were performed prior IVT injections (Day 0) and at the termination of the study (Day 7).

### Eye cup cross-sections

Whole eyes were enucleated and fixed in 4% paraformaldehyde (PFA) for an over-night period at 4 °C. Following fixation, the tissues underwent a 15-min wash in 1 × Tris-Buffered Saline (TBS) at room temperature, and eye cups were then carefully pre-pared. To prevent the formation of ice crystals, the tissues were further rinsed in a series of sucrose solutions at concentrations of 10%, 20%, and 30%. Eye cups then were placed in optimal cutting temperature (O.C.T.) compound (SAK4583, Sakura), frozen, and sectioned at 7 μm thickness. Hematoxylin and eosin (H&E) staining was performed on these sections, and images were captured using a stereo microscope (DM6 B, Leica Microsystems, Germany).

### Immunohistochemistry of retinal flat-mounts

The eyes were enucleated and fixed in 4% PFA solution for 30 min at room temperature. Subsequently, retinas were dissected using fine forceps and four radial incisions were made to flatten the retina. The flattened retinas were then fixed in 4% PFA overnight at 4 °C. After fixation, the tissues were washed in 1 × TBS for a duration of 5–6 h at 4 °C and subjected to blocking with a solution containing 10% goat serum and 0.5% Triton in 1 × TBS. After blocking retinal flat-mounts were washed with washing solution (1% goat serum + 0.1% Triton in 1 × TBS) and incubated with following primary antibodies: rabbit anti-Iba-1 (dilution 1:200, cat. no. 019–19,741, WAKO), and mouse anti-glial fibrillary acid-ic protein (GFAP) (1:1000, G3893, Sigma Aldrich) overnight at 4 °C. On the next day the tissues were washed with washing solution and incubated with secondary antibodies: anti rabbit Alexa Fluor (AF) 594 (1:500, A32740, Invitrogen) and anti-mouse AF488 (1:500, A21121, Invitrogen). Flat-mounts were washed again with washing solution and counter-stained with DAPI (1:10 000, 6843.1, Carl Roth), washed and mounted with Fluoroshield (F6937-20ML, Sigma Aldrich).

Slides were imaged using a stereo microscope (DM6 B, Leica Microsystems, Germany). For Iba-1 analysis twelve images were taken from each retinal flat-mount at 12 different locations (4 in periphery, 4 in middle and 4 in the central part). Both Iba-1 active and silent cells were manually counted for each image, using Image J software (v1.46r, National Institute of Health, USA). GFAP-positive cells were qualitatively assessed by visual inspection of stained flat-mount images.

### Data analysis

Statistical analysis was performed using GraphPad Prism (version 10.0.2, GraphPad Software, USA). Data were analyzed using one-way or two-way ANOVA followed by Dunnett’s or Šídák's multiple comparisons tests and presented as mean ± standard error of mean (SEM). Significance was determined according to the p value: *, *p* < 0.05, **, *p* < 0.01, ***, *p* < 0.001, and ****, *p* < 0.0001.

## Supplementary Information

Supplementary Table S1.

## Data Availability

All fERG a-wave and b-wave absolute values can be found in Supplementary Table S1. Other datasets used and/or analyzed during the current study are available from the corresponding author upon reasonable request.
