# Correction: Prospects and limitations of cumate-inducible lentivirus as a tool for investigating VEGF-A-mediated pathology in diabetic retinopathy

**DOI:** 10.1038/s41598-025-17850-0

**Published:** 2025-09-25

**Authors:** Inesa Lelyte, Vidhya R. Rao, Giedrius Kalesnykas, Symantas Ragauskas, Simon Kaja, Zubair Ahmed

**Affiliations:** 1 Institute of Inflammation and Ageing, University of Birmingham, Edgbaston, Birmingham, B15 2TT UK; 2 R&D Division, Experimentica Ltd., 10243 Vilnius, Lithuania; 3 Department of Ophthalmology, Loyola University Chicago, Maywood, IL 60153 USA; 4 R&D Division, Experimentica Ltd., Kuopio, Finland; 5 Experimentica Inc., Fort Worth, TX USA; 6 Department of Molecular Pharmacology and Neuroscience, Loyola University Chicago, Maywood, IL 60153 USA; 7 Centre for Trauma Sciences Research, University of Birmingham, Edgbaston, Birmingham, B15 2TT UK

Correction to: *Scientific Reports* 10.1038/s41598-024-63590-y, published online 21 June 2024

The original version of this Article contained an error in Figure 4, where the ‘Baseline Cumate 0.6 mg/2 μL’ panel was a duplication of the ‘3 days after IVT Control’ panel.

The original Figure 4 and accompanying legend appear below.

**Fig. 4:**
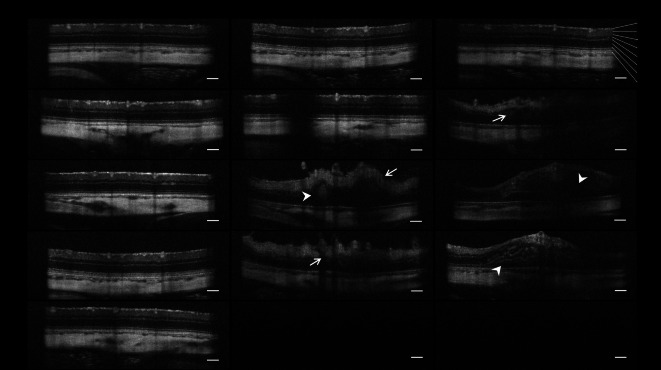
IVT injection of cumate induced retinal degeneration in rat eyes. Cumate (administered at doses of 0.1 mg/2 µL; 0.2 mg/2 µL; 0.6 mg/2 µL, and 1.5 mg/5 µL) resulted in pathological retinal layering (arrows), accumulation of fluid within retinal layers, retinal hemorrhages (arrowheads), and the presence of vitreous opacities (bottom row) which completely prevented SD-OCT imaging on Day 3 and Day 7 post-IVT injection. NFL = nerve fiber layer; GCL = ganglion cell layer; IPL = inner plexiform layer; INL = inner nuclear layer; OPL = outer plexiform layer; ONL = outer nuclear layer; ELM = external limiting membrane; RPE = retinal pigmented epithelium. Scale bar = 200 µm.

The original Article has been corrected.

