# Supplementary material for: Prospects and limitations of cumate-inducible lentivirus as a tool for investigating VEGF-A-mediated pathology in diabetic retinopathy
Source: Sci Rep. 2024 Jun 21;14:14325. doi: 10.1038/s41598-024-63590-y (PMC11192717; doi:10.1038/s41598-024-63590-y)
Supplement: Supplementary file 1 — Supplementary Table S1. [file 41598_2024_63590_MOESM1_ESM.docx]

**SUPPLEMENTARY MATERIAL**

**Prospects and limitations of cumate-inducible lentivirus as a tool for investigating VEGF-A-mediated pathology in diabetic retinopathy**

Inesa Lelyte^1,2,3,*^, Vidhya R. Rao^3^, Giedrius Kalesnykas^2,4^, Symantas Ragauskas^2^, Simon Kaja^3,5^, Zubair Ahmed^1,6,*^

^1^Institute of Inflammation and Ageing, University of Birmingham, Edgbaston, Birmingham, B15 2TT, UK

^2^R&D Division, Experimentica Ltd., 10243 Vilnius, Lithuania; [symantas.ragauskas@experimentica.com](mailto:symantas.ragauskas@experimentica.com)

^3^Department of Ophthalmology, Loyola University Chicago, Maywood, IL 60153, USA; [vrao2@luc.edu](mailto:vrao2@luc.edu)

^4^R&D Division, Experimentica Ltd., Kuopio, Finland, and Experimentica Inc., Fort Worth, TX, USA; [giedrius.kalesnykas@experimentica.com](mailto:giedrius.kalesnykas@experimentica.com)

^5^Department of Molecular Pharmacology & Neuroscience, Loyola University Chicago, Maywood, IL 60153, USA; [skaja@luc.edu](mailto:skaja@luc.edu)

^6^Centre for Trauma Sciences Research, University of Birmingham, Edgbaston, Birmingham, B15 2TT, UK

***** **Corresponding authors:**

Professor Zubair Ahmed, Institute of Inflammation and Ageing, University of Birmingham, Edgbaston, Birmingham, B15 2TT, UK; Tel: +44 121 4148854; Email: [z.ahmed.1@bham.ac.uk](mailto:z.ahmed.1@bham.ac.uk) and Inesa Lelyte, R&D Division, Experimentica Ltd., 10243 Vilnius, Lithuania. Email: [inesa@experimentica.com](mailto:inesa@experimentica.com)

**ORCID IDs:** 0000-0001-6267-6442 (Z.A); 0000-0001-6878-521X (S.K); 0000-0003-0228-1689 (V.R.R); 0000-0002-9014-3165 (I.L); 0000-0003-2172-7164 (G.K)

**Supplementary material**

Table S1. fERG a-wave and b-wave amplitude absolute values, difference and Šídák's multiple comparisons test results of all treatment groups at stimulus intensities -3.6, -2.6, -0.6, 0.4, 0.6 [log (cd.s.m-2)] (Baseline vs. Day 7). Data are presented as mean ± SEM.

| **a-wave amplitude, µV** | | | | | |
| --- | --- | --- | --- | --- | --- |
| **Stimulus intensity** | **-3.6** | **-2.6** | **-0.6** | **0.4** | **0.6** |
| **Control** | | | | | |
| Baseline |  |  | 123.97 ± 14.32 | 231.95 ± 12.31 | 233.55 ± 14.62 |
| Day 7 |  |  | 157.2 ± 7.69 | 270.73 ± 7.52 | 282.29 ± 10.78 |
| Difference, µV |  |  | 33.23 | 38.78 | 48.74 |
| Difference % |  |  | 26.80 | 16.72 | 20.87 |
| Summary |  |  | ns | ***** | ****** |
| *p* value |  |  | 0.07 | **< 0.05** | **< 0.01** |
|  | | | | | |
| **Cumate 0.6 mg/2 µL** | | | | | |
| Baseline |  |  | 138.31 ± 16.79 | 240.56 ± 9.49 | 240.75 ± 9.68 |
| Day 7 |  |  | 108.91 ± 12.51 | 173.8 ± 18.94 | 187.19 ± 18.85 |
| Difference, µV |  |  | -29.4 | -66.76 | -53.56 |
| Difference % |  |  | -21.26 | -27.75 | -22.25 |
| Summary |  |  | ns | ****** | ***** |
| *p* value |  |  | 0.48 | **< 0.01** | **< 0.05** |
|  | | | | | |
| **Cumate 1.5 mg/5 µL** | | | | | |
| Baseline |  |  | 139.07 ± 6.88 | 238.45 ± 13.56 | 244.12 ± 15.41 |
| Day 7 |  |  | 5.04 ± 3.62 | 10.76 ± 7.31 | 12.4 ± 8.45 |
| Difference, µV |  |  | -134.03 | -227.69 | -231.72 |
| Difference % |  |  | -96.38 | -95.49 | -94.92 |
| Summary |  |  | ******* | ******* | ******* |
| *p* value |  |  | **< 0.001** | **< 0.001** | **< 0.001** |
| **b-wave amplitude, µV** | | | | | |
| **Stimulus intensity** | **-3.6** | **-2.6** | **-0.6** | **0.4** | **0.6** |
| **Control** | | | | | |
| Baseline | 56.91 ± 3.81 | 157.16 ± 13.9 | 291.01 ± 24.32 | 398.25 ± 30.19 | 433.7 ± 27.17 |
| Day 7 | 61.41 ± 11.79 | 195.96 ± 13.69 | 340.83 ± 24.41 | 483.33 ± 29.81 | 508.56 ± 24.62 |
| Difference, µV | 4.5 | 38.8 | 49.82 | 85.08 | 74.86 |
| Difference % | 7.91 | 24.69 | 17.12 | 21.36 | 17.26 |
| Summary | ns | ns | ns | ***** | ns |
| *p* value | > 0.99 | 0.62 | 0.35 | **< 0.05** | 0.06 |
|  | | | | | |
| **Cumate 0.6 mg/2 µL** | | | | | |
| Baseline | 53.95 ± 6.04 | 170.66 ± 8.91 | 294.53 ± 21.89 | 404 ± 21.27 | 418.38 ± 17.88 |
| Day 7 | 24.01 ± 9.95 | 83.64 ± 17.71 | 153.08 ± 20.83 | 232.72 ± 26 | 252.02 ± 26.41 |
| Difference, µV | -29.94 | -87.02 | -141.45 | -171.28 | -166.36 |
| Difference % | -55.50 | -50.99 | -48.03 | -42.40 | -39.76 |
| Summary | ns | ****** | ******* | ******* | ******* |
| *p* value | 0.78 | **< 0.01** | **< 0.001** | **< 0.001** | **< 0.001** |
|  | | | | | |
| **Cumate 1.5 mg/5 µL** | | | | | |
| Baseline | 54.57 ± 9.71 | 181.03 ± 10.8 | 325.63 ± 27.47 | 443.16 ± 37.16 | 464.7 ± 35.8 |
| Day 7 | 4.38 ± 4.38 | 7.86 ± 7.86 | 9.06 ± 6.33 | 14.51 ± 9.82 | 22.69 ± 15.36 |
| Difference, µV | -50.19 | -173.17 | -316.57 | -428.65 | -442.01 |
| Difference % | -91.97 | -95.66 | -97.22 | -96.73 | -95.12 |
| Summary | ns | ******* | ******* | ******* | ******* |
| *p* value | 0.26 | **< 0.001** | **< 0.001** | **< 0.001** | **< 0.001** |
